# Effect of sodium thiosulfate on preventing renal ischemia-reperfusion injury in high-fat diet-fed rats: the role of renal mitochondrial quality

**DOI:** 10.1186/s40659-025-00636-z

**Published:** 2025-08-18

**Authors:** Priyanka N. Prem, Gino A. Kurian

**Affiliations:** 1 School of Chemical and Biotechnology, SASTRA Deemed University, Tirumalaisamudram, Thanjavur, Tamil Nadu India; 2 Vascular Biology Lab, SASTRA Deemed University, Tirumalaisamudram, Thanjavur, Tamil Nadu India; 3 Vascular Biology Lab, School of Chemical and Biotechnology, SASTRA Deemed University, Thanjavur, India

**Keywords:** Renal ischemia reperfusion, Sodium thiosulfate, High fat diet, Mitochondria, Oxidative stress

## Abstract

**Objective:**

Sodium Thiosulfate (STS), a clinically approved agent for cyanide poisoning and vascular calcification, possesses antioxidant, anti-inflammatory, mitochondrial preservation, and metal chelation capabilities, rendering it a promising candidate for managing ischemia-reperfusion (IR) injury. The detrimental impact of high-fat diets (HD) on the outcomes of IR during renal surgeries is well-documented. However, the potential of STS to ameliorate renal IR injury in rat fed with high fat diet is not known.

**Methods:**

Male Wistar rats were fed a standard diet (SD) or a high-fat diet (HD) for 16 weeks before undergoing an IR protocol (45 min of ischemia followed by 24 h of reperfusion). STS (10 mg/kg) was administered 30 min before IR.

**Results:**

STS effectively mitigated IR-induced physiological decline and tissue damage in SD rats but was less effective in HD rats. To explore this difference, we measured renal mitochondrial quality. STS improved mitochondrial bioenergetics, balanced mitochondrial dynamics, and increased mitochondrial copy number in SD-IR rats more than in HD-IR rats. Additionally, STS significantly reduced oxidative stress and upregulated Pgc-1α, Polg, and Tfam genes in SD-IR rats but had a lesser effect in HD-IR rats. The 16-week HD significantly reduced renal mitochondrial quality at the basal level, hindering STS-mediated protection.

**Conclusion:**

These findings highlight the efficacy of STS in managing renal IR and emphasize the need for nutritional support to restore mitochondrial function in high-fat diet subjects.

**Supplementary Information:**

The online version contains supplementary material available at 10.1186/s40659-025-00636-z.

## Introduction

Ischemia-reperfusion (IR) injury is a common complication in renal transplantation and conditions like sepsis, infarction, renal artery stenosis, and vascular lesions [1, 2].IR is a leading cause of acute kidney injury (AKI) and remains a significant unmet medical need [3]. There is an urgent need for effective therapies targeting AKI [4].

Mitochondria play a crucial role in AKI pathogenesis, with early studies showing mitochondrial dysfunction can occur before AKI symptoms and serve as a target for mitigating IR injury [5]. This dysfunction results from defects in mitochondrial structure, dynamics, and biogenesis during AKI development, contributing to AKI progression and the transition to chronic kidney disease (CKD) [5–8]. Renal IR injury involves altered hemodynamics, tubular damage, congestion, and inflammation, with key mediators generated in mitochondria, the nucleus, and the endoplasmic reticulum [1, 9]. Previous studies have reported reduced mitochondrial content, structural alterations, swelling, and changes in membrane potential in IR-affected tissues [10–12].

Early studies have shown that a high-fat diet (HD) negatively impacts renal diseases, accelerating their progression. HD can impair renal function by altering cellular mitochondria, compromising the kidney’s ability to recover from ischemic challenges during reperfusion [13, 14]. HD also increases oxidative stress and induces mitochondrial fission in tubular cells, promoting apoptosis. Research suggests that HD adversely affects renal function by disrupting Wnt/β-catenin signaling, activating protease-activating receptor 2 (PAR2), and interfering with other reno-protective mechanisms, all linked to mitochondrial function [15, 16].

Given mitochondria’s key role in AKI, particularly in IR injury, targeting them has become a major research focus [10, 12]. Strategies for managing experimental AKI often involve protecting cardiolipin, preventing mitochondrial fragmentation, promoting mitochondrial biogenesis, and inhibiting mPTP opening and mitochondrial oxidant production [17–19].

Sodium thiosulfate (STS), an antioxidant and calcium chelator, is known to protect cardiac tissue from IR injury by preserving mitochondrial function and activating pro-survival pathways like PI3K, Akt, and JAK-STAT [20–23]. Some studies have also demonstrated STS’s renal protective effects against urolithiasis and vascular calcification [24–26]. However, its effectiveness in mitigating IR-induced AKI, particularly in rats under stress from high-fat diet consumption, is not well studied and is the focus of this research.

## Methodology

### Animals

Male Wistar rodents weighing between 120 and 150 g were obtained from the Central Animal Facility of SASTRA University. Prior to the experiments, animals were left to acclimatise for one week under standard conditions of temperature (22 ± 1 °C), humidity (55 ± 5%), and 12 h/12 h light/dark cycles with unrestricted access to food and water.

### Animal groups and surgical procedure

The animal experiments were conducted in accordance with the Committee for Control and Supervision of Experiments on Animals (CCSEA), India, and approved by the Institutional Animal Ethical Committee (IAEC) at SASTRA Deemed University, Thanjavur (257/SASTRA/IAEC/RPP). All procedures followed ARRIVE guidelines. Thirty-six male Wistar rats were divided into two groups (*n* = 18 per group): standard diet-fed (SD) and high-fat diet-fed (HD) (sample size determined by GPower 3.1). The SD group received a cereal-based diet with 395 kcal/100 g, while the HD group received a high-fat diet with 540 kcal/100 g for 16 weeks after weaning.

Following the induction phase, the animals were surgically operated. Meloxicam was used for analgesia and isoflurane for anaesthesia. Rats were put on a heating pad at 37.5 ± 1 °C and examined for induction by pinch stimulation after being anaesthetised with 1–4% isoflurane in oxygen prior to surgery. Each kidney’s vascular pedicles were mobilised, and the renal pedicles were blocked using non-traumatic mini-bulldog clamps. The clamps were taken off to enable renal reperfusion following the ischaemic phase. Before suturing, saline was infused into the peritoneal cavity to control fluid loss. In order to prevent sepsis, povidone-iodine and neosporin were applied to the sutured area. Meloxicam (1 mg/kg) was injected subcutaneously to relieve pain.

#### Groups

After 16 weeks, each main group was divided into three subgroups (*n* = 6): (1) Sham: Renal pedicles were exposed for 45 min without clamping; (2) Ischemia-reperfusion (IR): Renal pedicles were clamped for 45 min to induce ischemia, followed by 24 h of reperfusion; (3) STS pre-treatment (S): STS (10 mg/kg) was administered intraperitoneally 15 min before 45 min of ischemia and 24 h of reperfusion (dose determined through a pilot study). Rats were euthanized after reperfusion with an isoflurane overdose, and kidneys were collected for biochemical and molecular analysis. Three kidneys per group were kept in formalin for hematoxylin/eosin histopathological analysis. The following pathologies were used to evaluate the pathomorphological lesions under a microscope based on the EGTI (Endothelium, Glomeruli, Tubular/interstitial) scoring system: glomerular congestion, tubular dilatation, tubular degeneration, tubular necrosis, and the presence of eosinophilic casts. Based on the severity, the changes were rated on a 4-point scale (0–nil/absent, 1-mild, 2-moderate, 3-marked, 4-severe), and the average score is provided [14].

### Biochemical parameters

Blood samples were collected from the tail vein both before and after the surgery, separated into plasma using a centrifuge set up at 3000 rpm for 10 min, and then stored at -80 °C for additional examination. Diagnostic kits (Agappa Diagnostics Ltd. ,India)were used to measure the amounts of BUN and plasma creatinine. For urine collection, the animals were placed in metabolic cages following ischemia. Urine samples were collected at intervals of 4 h, 8 h, and 24 h post-surgery. The total urine volume was measured at the end of the 24-hour period. Creatinine, and BUN levels were measured using diagnostic kits. Urinary KIM-1 levels were analyzed with an ELISA kit (Krishgen Biosystems, Mumbai, India) following the manufacturer’s instructions.

### Creatinine clearance rate

Creatinine clearance (CrCl) calculation performed using the equation by Pestel et al. (2007) [27]: Creatinine clearance = (1000 ⁎ urine volume ⁎ concentration of creatinine in urine)/ concentration of creatinine in serum.

### Na+/K + ATPase activity

Na-K ATPase activity was estimated using 0.5 mg/ml renal homogenate in a reaction mixture containing (in mM) NaCl: 140, KCl: 14, MgCl_2_: 3, Tris-HCl, pH 7.4, Na_2_-ATP: 30. After 2 h of incubation at 37 °C, the samples were removed and the reaction was terminated by the addition of 2 ml of 10% (w/v) trichloroacetic acid. Using Fiske and Subba Row’s method [28]– [29], the amount of inorganic phosphate formed was measured.

### Caspase 3 activity

In Tris-HCl buffer (pH 8) containing 400 mM NaCl and 25 mM EDTA, renal tissue homogenate was prepared. Using a caspase-3 fluorogenic substrate (Sigma Aldrich, USA), caspase-3 activity was quantified spectrophotometrically. The tissue homogenate was mixed with a solution that contained substrate (25 µM), glycerol (20%), EDTA (0.5 mM), DTT (5 mM), and HEPES (100 mM). For sixty minutes, the enzyme activity was observed at 37 °C at an ex/em- 365/465 nm [30].

### TUNEL staining

TUNEL (terminal deoxynucleotidyl transferase mediated dUTP nick-end labelling) study of renal tissue positive for DNA strand breaks was carried out using fluorescence microscopy and a fluorescence detection reagent (Takara Bio Inc., Japan) [31].

### Mitochondrial isolation

In accordance with Afanasyeva et al. (2018), rat kidney mitochondria were isolated using the differential centrifugation technique [32]. As described below, isolated mitochondrial function was analysed.

### Antioxidant enzymes and lipid peroxidation

The concentration of thiobarbituric acid reactive substances (TBARS) and glutathione (GSH) as well as the activities of glutathione reductase (GR), superoxide dismutase (SOD), and catalase enzymes were measured in the mitochondrial samples according to the previously described methods [20].

#### Mitochondria evaluation

- Activity of electron transport chain enzymes, namely Rotenone sensitive NADH-oxidoreductase (NQR), succinate decylubiquinone DCPIP reductase (SQR), ubiquinol cytochrome-c reductase (QCR), and cytochrome c oxidase (COX), analysed spectrophotometrically according to the previously described method [30].
- ATP concentration and ATP production capability: Based on the luminescence produced by the reaction of ATP with the substrates luciferase and -luciferin, the ATPlite kit (Perkin Elmer, Massachusetts, United States) was utilised to measure ATP levels in the isolated mitochondria of all groups. In the presence of glutamate/malate (5/2.5 mM) and succinate (2.5 mM) energised medium, the ability to produce ATP was evaluated and compared to the ATP level under non-energized conditions [30].
- Gene expression: From the renal tissues, mRNA was extracted using TRIzol reagent (15596026, Thermo Scientific, USA), and cDNA conversion was performed using kit based method. Gene expression was measured based on Sybr green chemistry (F415, Thermo Scientific) using qPCR (ABI7500, Thermo Scientific, USA). The expression of nuclear-encoded β actin, Tfam, Polg, Pgc 1α, Fis 1, Mff, Dnm 1, Mfn 1, Mfn 2, Opa 1, Pink 1, Parkin, and Optn were analysed in rat kidney samples.

For the estimation of mitochondrial copy number, DNA was isolated from renal tissue samples using phenol-chloroform-isoamyl alcohol (Himedia, Mumbai) method according to manufacturer’s instruction (Himedia, Mumbai).The expression for the mitochondrial encoded ND1 (Mt-ND1) and nuclear-encoded β actin were analyzed in total DNA using qPCR (ABI7500, Thermo Scientific, Massachusetts, USA) and the ratio of the relative gene expression of the Mt-ND1and β actin gene was calculated and presented as mitochondrial DNA copy number [13].

The calculation of gene expression followed the method of Livak and Schmittgen [33]. The primer sequence for each gene is shown in Supplementary Table 1.

### Statistical analysis

All data are presented as mean ± standard deviation (SD). Using a one-way analysis of variance, followed by a post hoc test, intergroup comparisons were conducted. Dunnet’s test was used to ascertain the difference between the groups, and *p* < 0.05 was regarded as statistically significant.

## Results

### Administration of STS improved renal physiological recovery from 45 min bilateral renal artery ligation

**Fig. 1:**
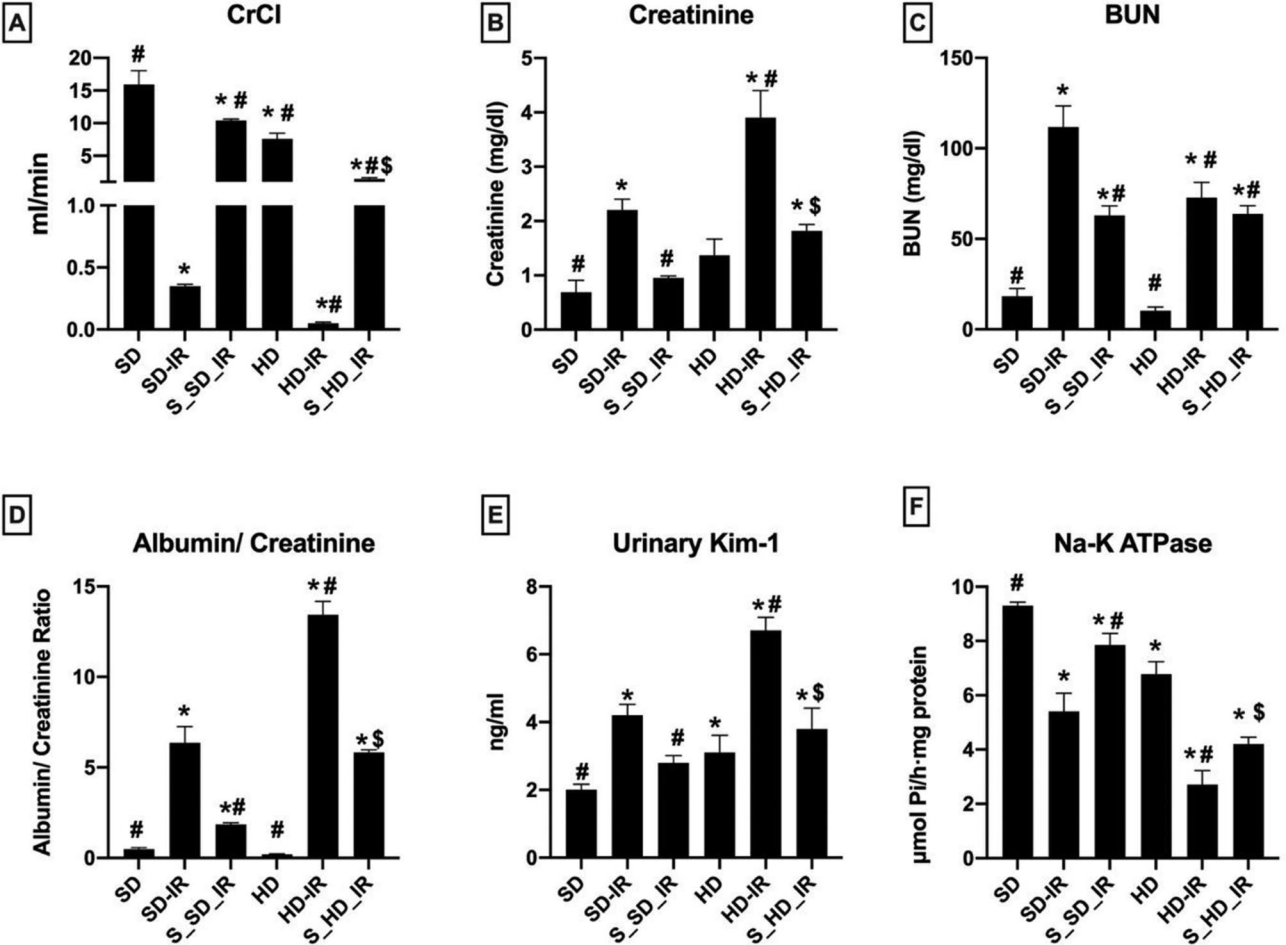
STS impact on the renal physiology and functional analysis of SD and HD fed rats subjected to IR injury. Renal functional markers (**A**) Creatinine clearance (CrCl), (**B**) Plasma creatinine, (**C**) Plasma BUN, and (**D**) Urinary albumin/Creatinine ratio (**E**) Urinary kim-1 level and (**F**) Na-K ATPase activity. *p < 0.05 vs. SD, ^**#**^ *p* < 0.05 vs. IR, ^**$**^*p* < 0.05 in S_HD-IR vs. HD-IR. The data are presented as mean ± SD (*n* = 6/group). SD-Standard diet, HD- High fat diet, IR-Ischemia-reperfusion, S- STS Pre-treatment

Compared with the normal renal sham, the reperfused kidneys from SD and HD exhibited decreased levels of CrCl, increased creatinine and BUN levels in plasma, elevated albumin/creatinine ratio and kim-1 levels in urine and reduced renal Na-K ATPase activity were observed, but with higher degree of damage was noted in HD_IR (Fig. 1). These changes were significantly improved with STS pre-treatment in both SD and HD rats (Fig. 1). Accordingly, the figure display the improvement in creatinine clearance rate, urine output, decreased blood urea nitrogen and creatinine level with subsequent increase in Na-K ATPase activity by STS pre-treatment to renal IR. From respective IR control, CrCl values were increased by 96% in both S_SD-IR and S_HD-IR. Similarly blood urea nitrogen and creatinine level were significantly increased in IR control (SD-IR: by 83% and 68% from SD, HD-IR: by 85% and 64% from HD) was reduced substantially in S_SD-IR (42% and 56%) and S_HD-IR (12% and 53%). The renal tissue physiological recovery from reperfusion damage was further evident from the improved enzyme activities of Na-K ATPase (percentage declined activity from normal: SD-IR- 41%, HD-IR- 60%, S_SD-IR- 15% and S_HD-IR- 38%) and reduced albumin/creatinine ratio (percentage increase from normal: SD-IR = 92%, HD-IR- 98%, S_SD-IR- 74% and S_HD-IR- 96%) in the STS treated groups (Fig. 1).

### Alterations in renal histology associated with IR reduced significantly by STS treatment

**Fig. 2:**
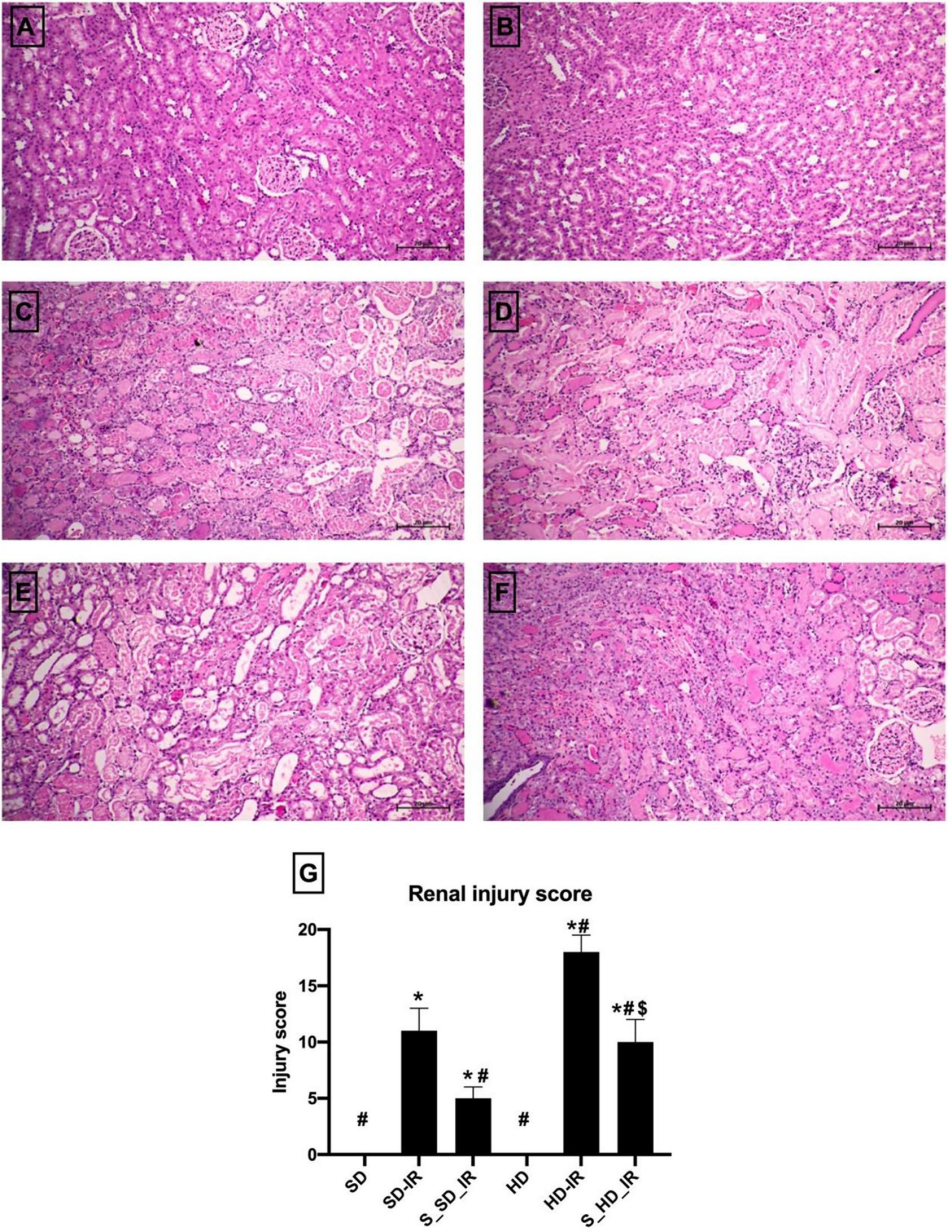
STS impact on the IR injury in renal tissue from SD and HD fed rats. Representative H&E stained images for (**A**) SD (**B**) HD, (**C**) SD-IR (**D**) HD-IR, (**E**) S_SD-IR, and (**F**) S_HD-IR (The images are presented at 10x magnification, scale bar- 20 μm) and (**G**) Renal injury score from H and E staining (*n* = 3) *p < 0.05 vs. SD, ^**#**^ *p* < 0.05 vs. IR, ^**$**^*p* < 0.05 in S_HD-IR vs. HD-IR. The data are presented as mean ± SD (*n* = 3/group). SD-Standard diet, HD- High fat diet, IR-Ischemia-reperfusion, S- STS Pre-treatment

**Fig. 3:**
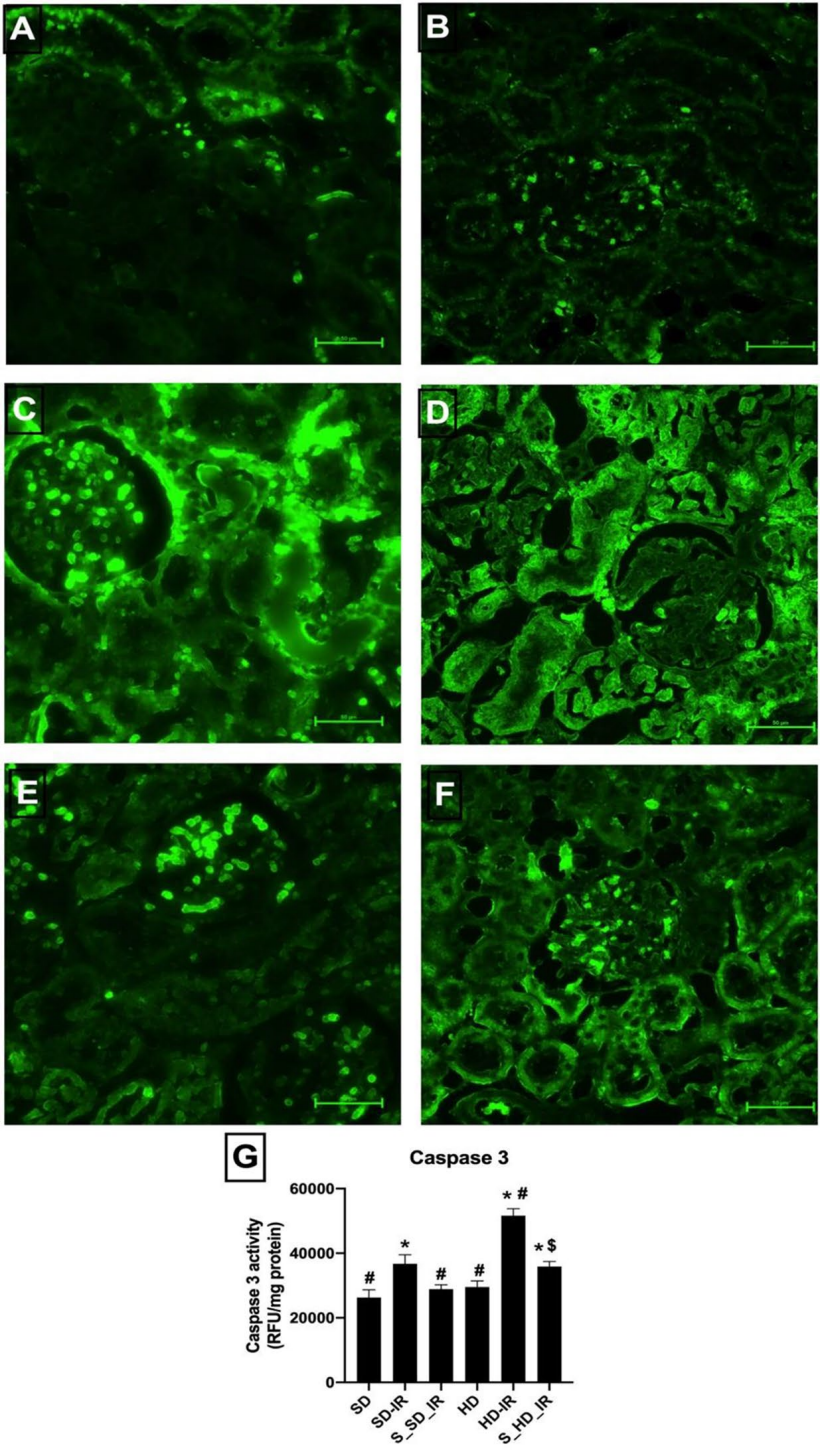
STS impact on the cell death due to IR injury in renal tissue from SD and HD fed rats. Representative TUNEL images for (**A**) SD (**B**) HD, (**C**) SD-IR (**D**) HD-IR, (**E**) S_SD-IR, and (**F**) S_HD-IR (The images are presented at 40x magnification, scale bar- 50 μm) and renal cell death analysis by (**G**) caspase 3 activity.*p < 0.05 vs. SD, ^**#**^ *p* < 0.05 vs. IR, ^**$**^*p* < 0.05 in S_HD-IR vs. HD-IR. The data are presented as mean ± SD (*n* = 6/group). SD-Standard diet, HD- High fat diet, IR-Ischemia-reperfusion, S- STS Pre-treatment

H&E staining data demonstrated that SD-IR and HD-IR kidneys displayed prominent structural changes compared to the normal, when subjected to bilateral clamping of renal artery for 45 min and subsequent reperfusion for 24 h (Fig. 2). IR in HD rat kidney enhanced the tubular damage and necrosis, increased glomerular congestion and haemorrhage compared to SD (Injury score: SD-IR- 11 ± 2, HD-IR- 18 ± 2) (Fig. 2G). Similar to the normal animals, STS treatment reversed the IR associated structural alterations in HD animals, thereby reduced significantly the injury score (S_SD-IR- 5 ± 1, S_HD-IR- 10 ± 2) (Fig. 2E and F). Furthermore, the apoptotic injury measured by using TUNEL kit showed significant elevation in TUNEL positive cells in both SD_IR and HD_IR renal tissues and was effectively reduced by the STS treatment in both experimental groups (Fig. 3A-F)). TUNEL results were supported by significant decline in caspase 3 activity in STS treated SD_IR and HD_IR renal tissues, where the decline was prominent in SD rats (Fig. 3G).

### STS reduced oxidative stress linked with IR in renal mitochondria

**Fig. 4:**
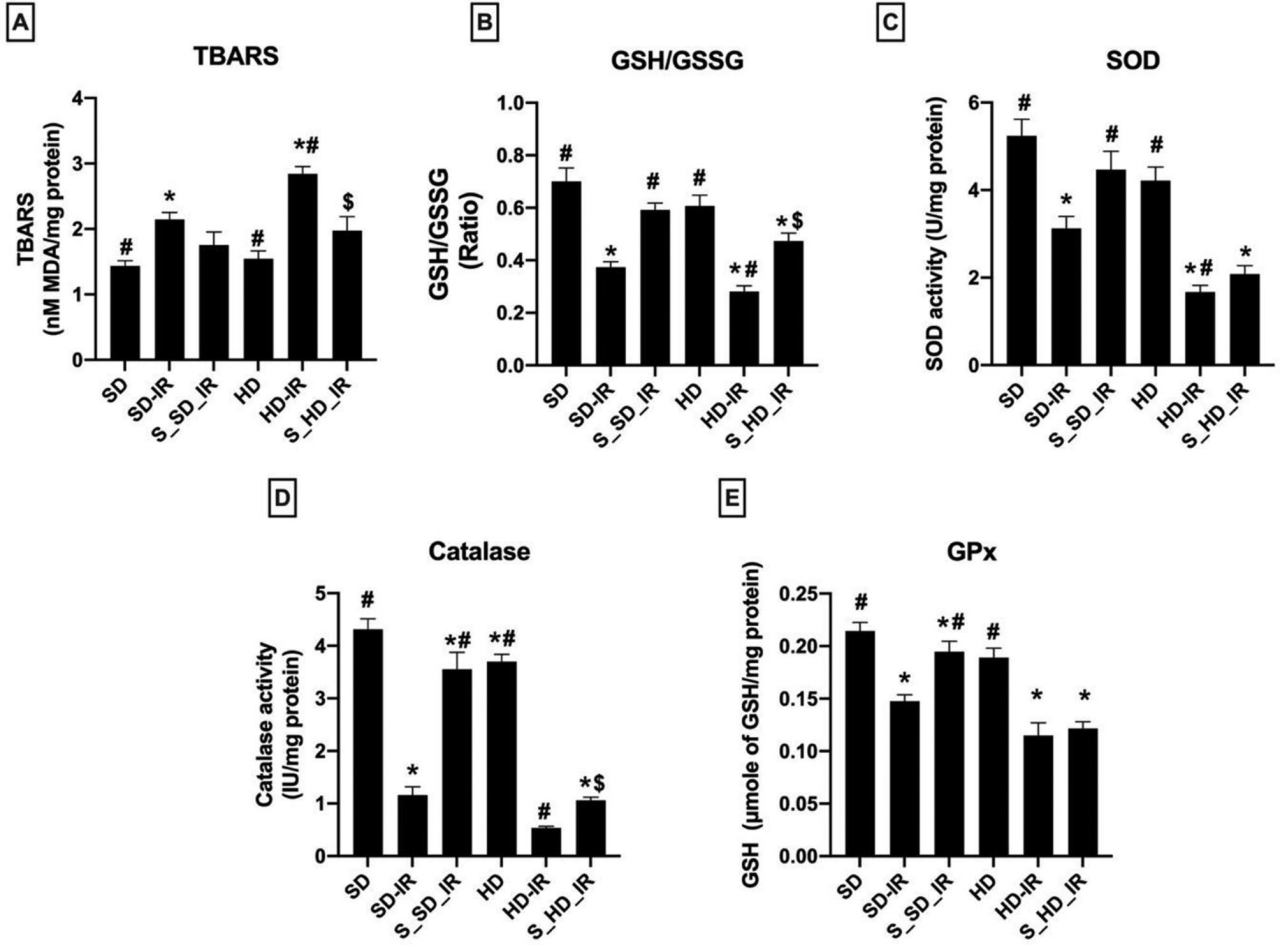
Impact of STS on the oxidative stress and antioxidants levels in the renal mitochondrial fraction of SD and HD treated rats subjected to IR injury. (**A**) Shows lipid peroxidation measured by TBARS, (**B**) GSH/GSSG ratio, (**C**) SOD activity, (**D**) Catalase activity, and (**E**) represents GPx activity. *p < 0.05 vs. SD, ^**#**^ *p* < 0.05 vs. IR, ^**$**^*p* < 0.05 in S_HD-IR vs. HD-IR. The data are presented as mean ± SD (*n* = 6/group). SD-Standard diet, HD- High fat diet, IR-Ischemia-reperfusion, S- STS Pre-treatment

Figure 4 shows the activities of antioxidant enzymes and lipid peroxidation level in the renal mitochondria from different experimental groups. Result demonstrated that SD and HD kidney mitochondria exhibited a significant increase in TBARS and decrease in the level of GSH/GSSG ratio upon IR induction indicate higher oxidative stress, when compared with the normal animal tissues (Fig. 4A-E). However, STS treated tissues, significantly reduced lipid peroxidation level and improved GSH/GSSG ratio in the mitochondrial samples in SD and HD kidney, where the both diets treated kidney showed comparable changes (Fig. 4). But, upon reperfusion, mitochondrial antioxidant defense (determined by SOD, catalase, and GPx activities) were severely impaired in SD (% reduction in the activity- SD_IR: Catalase- 73%, SOD- 38%, GPx- 33%), and HD (% reduction in the activity- HD_IR: Catalase- 85%, SOD- 60%, GPx- 38%) from their respective control group. Unlike in the normal kidney, whereas STS could able to provide only moderate improvement in the antioxidant status in the HD kidney against IR induced oxidative stress (% improvement in the activity compared to IR control- S_SD-IR: Catalase- 67%, SOD- 30%, GPx- 26% ; S_HD-IR: Catalase- 50%, SOD- 19%, GPx- 8%) (Fig. 4).

### STS improved mitochondrial bioenergetics and thereby reduced IR inflicted mitochondrial functional deterioration

**Fig. 5:**
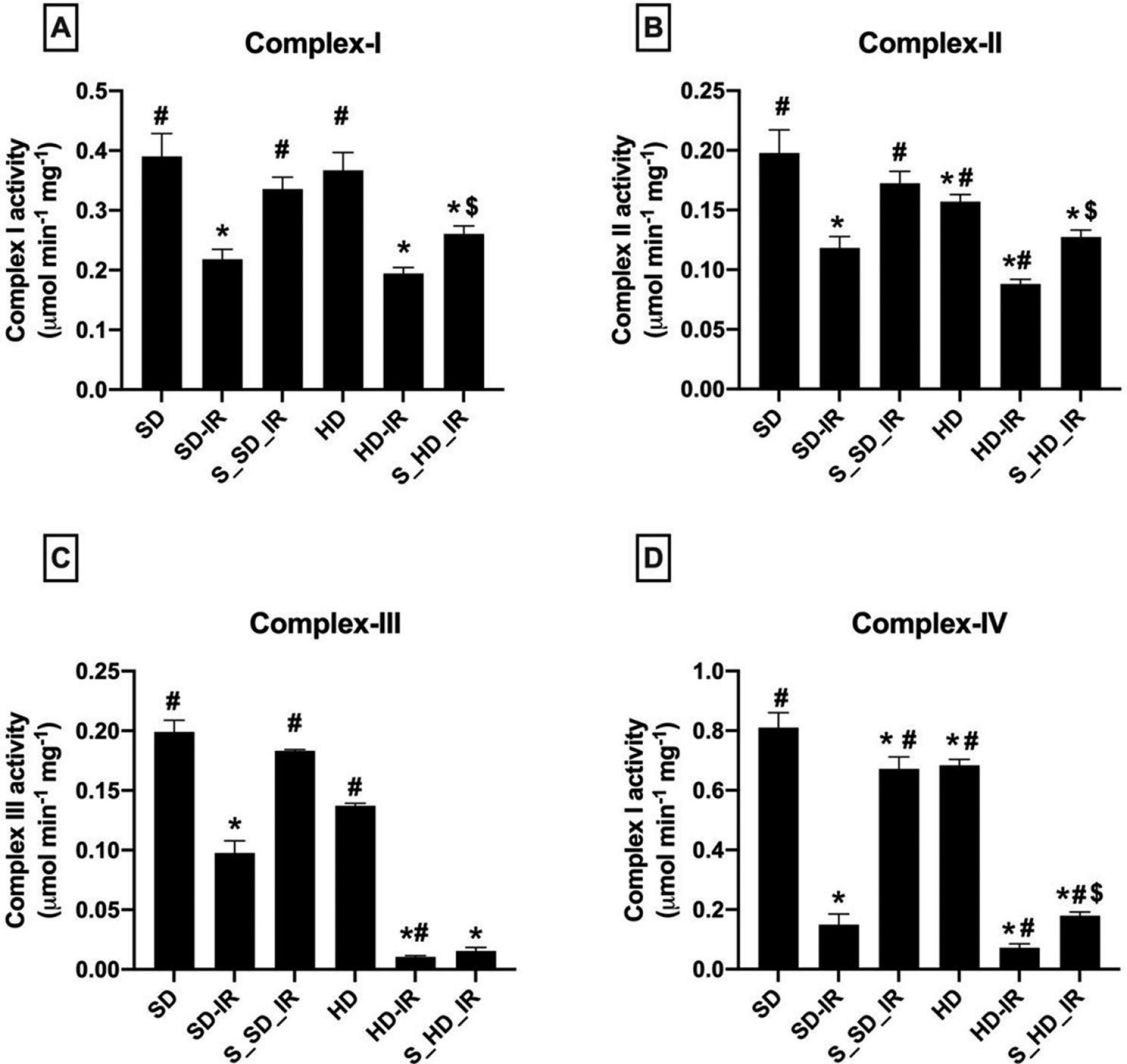
Impact of STS on the mitochondrial ETC enzymes in SD and HD treated rats subjected to IR injury. (**A**) Complex I activity, (**B**) Complex II activity, (**C**) Complex III activity and (**D**) Complex IV activity. Complex I activity was expressed as µmol NADH oxidized min-1 mg -1 protein; Complex II activity was expressed in µmol DCPIP reduced min-1 mg -1 protein; Complex III activity was expressed in µmol Cytochrome C reduced min-1 mg -1 protein; Complex IV activity was expressed in µmol Cytochrome C oxidized min-1 mg -1 protein. *p < 0.05 vs. SD, ^**#**^ *p* < 0.05 vs. IR, ^**$**^*p* < 0.05 in S_HD-IR vs. HD-IR. The data are presented as mean ± SD (*n* = 6/group). SD-Standard diet, HD- High fat diet, IR-Ischemia-reperfusion, S- STS Pre-treatment

As shown in Fig. 5, HD rats displayed deteriorated basal mitochondrial function, compared to the normal SD rats. Upon reperfusion, both SD and HD kidney showed a significant decline in the activities of mitochondrial ETC enzymes with a higher degree of decline in the HD_IR kidneys (Fig. 5A-D). STS pretreatment could effectively recover the normal and HD kidney from IR induced mitochondrial dysfunction but the recovery was better in SD-IR kidneys (% improvement from respective IR control- S_SD-IR: Complex I- 36%, Complex II- 30%, Complex III- 50%, Complex IV- 79%; S_HD-IR: Complex I- 26%, Complex II- 33%, Complex III- 32%, Complex IV- 58%).

**Fig. 6:**
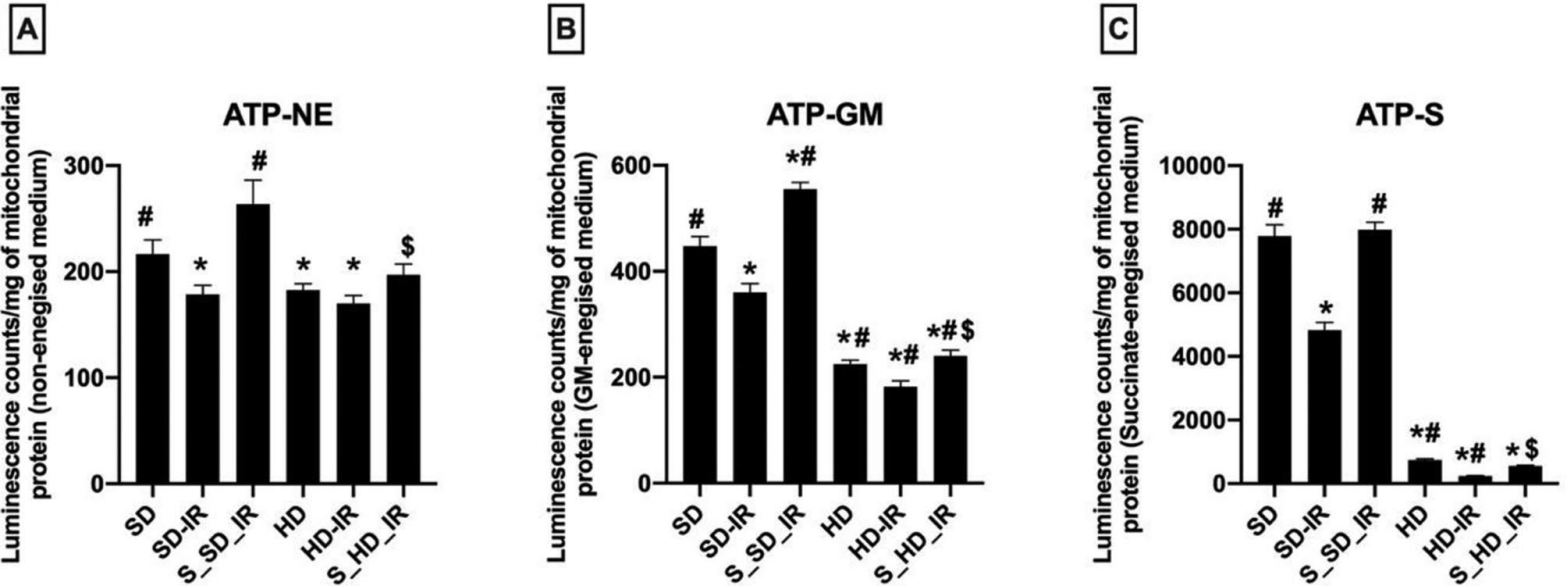
Analysis of changes in mitochondrial ATP production in STS pre-treated SD and HD rats subjected to renal IR. Graph represents measured ATP content using ATPlite luminescence kit, in (**A**) non-energized, (**B**) glutamate/malate energized and (**C**) succinate energized medium. *p < 0.05 vs. SD, ^**#**^ *p* < 0.05 vs. IR, ^**$**^*p* < 0.05 in S_HD-IR vs. HD-IR. The data are presented as mean ± SD (*n* = 6/group). SD-Standard diet, HD- High fat diet, IR-Ischemia-reperfusion, S- STS Pre-treatment

The ATP concentration in the isolated mitochondria from all experimental groups under non-energized and energized condition by utilizing GM (glutamate/malate) and succinate as substrate were measured and the results are shown in Fig. 6. Baseline ATP level under non-energized condition was found to be different among the SD and HD, where HD exhibited declined ATP concentration compared with SD (ATP: SD- 216 ± 13, HD- 182 ± 6). In energized condition, the ATP concentration in the HD kidney were decreased further (Fig. 6A-C). The decreased IR associated ATP concentration was increased by STS pre-treatment in both energized and non-energised conditions (Fig. 6).

**Fig. 7:**
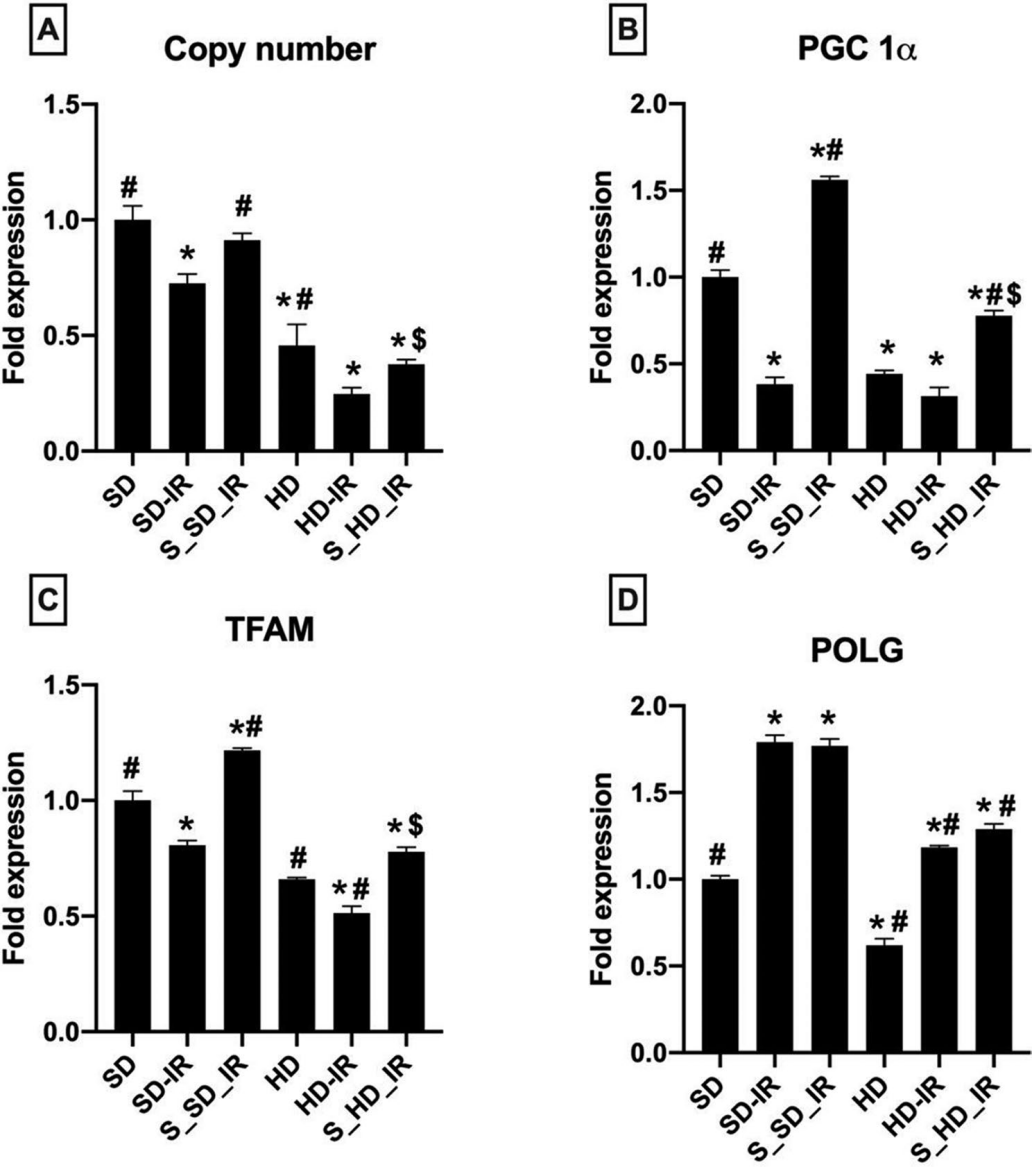
Gene expression analysis in in STS pre-treated SD and HD rats subjected to renal IR. Graph represents (**A**) mitochondrial copy number and (**B**) Pgc-1α expression (**C**) Tfam expression and (**D**) Polg in renal tissues. *p < 0.05 vs. SD, ^**#**^ *p* < 0.05 vs. IR, ^**$**^*p* < 0.05 in S_HD-IR vs. HD-IR. The data are presented as mean ± SD (*n* = 6/group). SD-Standard diet, HD- High fat diet, IR-Ischemia-reperfusion, S- STS Pre-treatment

To assess if the mitochondrial copy number plays a significant role in the reduced functional activity, we evaluated the mitochondrial copy number in the rat renal tissues from all the experimental groups and noted a significant (*p* < 0.05) reduction in the copy number in HD (0.75 ± 0.09)animal compared with SD (1 ± 0.06) in the basal level (Fig. 7). IR injury substantially decreased the copy number in both SD and HD (SD-IR: 0.72 ± 0.04, HD-IR- 0.24 ± 0.02), and pre-treatment of the animals with STS restored the copy number to nearly its own control levels. (Fig. 7A).

Further analysis of the expression level of Pgc-1α (Fig. 7B), which plays a critical role in the regulation of mitochondrial biogenesis and respiratory function (bioenergetics) showed a 56% (*p* < 0.05) decline in HD kidney. Interestingly, the IR injury caused drastic decline in the expression of Pgc-1α in SD rats than HD rats. STS pre-treatment shown to have biogenetic potential and improved the Pgc-1α expression by 75% and 59% from the respective IR groups. The mitochondrial transcription factor A (Tfam) is a nuclear-encoded protein that promotes the expression of the mitochondrial encoded genes. The IR induction severely decreased the gene expression levels of Tfam in SD and HD groups (Fig. 7C). Pre-treatment of kidney prior IR induction improved the Tfam levels only in both SD and HD fed rats. However, STS was found to exert minimal impact on the mitochondrial DNA polymerase γ (Polg), which is responsible for replication and repair of the mitochondrial genome (Fig. 7D).

### STS preserved the mitochondrial quality control events from IR challenge

**Fig. 8:**
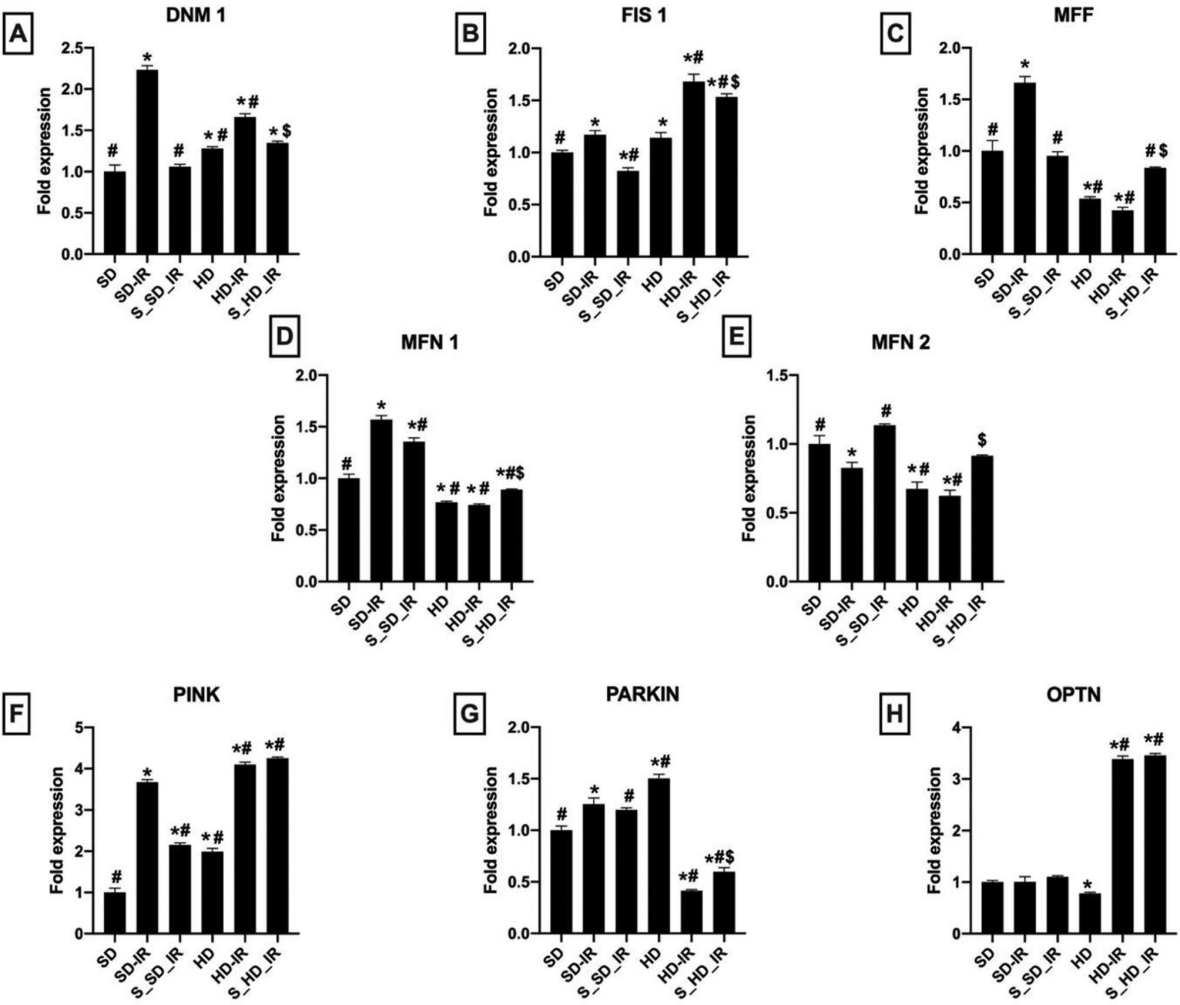
Impact of STS on the expression of genes encoding mitochondrial fission, fusion and mitophagy in renal tissues from SD and HD treated rats subjected to IR injury. The graph represents the mitochondrial fission genes (**A**) Dnm 1, (**B**) Fis 1, and (**C**) Mff; Mitochondrial fusion genes (**D**) Mfn 1 and (**E**) Mfn ; Mitophagic genes (**F**) Pink, (**G**) Parkin, and (**H**) Optn. *p < 0.05 vs. SD, ^**#**^ *p* < 0.05 vs. IR, ^**$**^*p* < 0.05 in S_HD-IR vs. HD-IR. The data are presented as mean ± SD (*n* = 6/group). SD-Standard diet, HD- High fat diet, IR-Ischemia-reperfusion, S- STS Pre-treatment

In general, dysfunctions in cellular bioenergetics are remedied by modulating mitochondrial dynamics. The expression of mitochondrial fission and fusion genes was therefore measured in the present study. Figure 8 demonstrates a decrease in Mfn 1 and Mfn 2 expression and increased Dnm 1 and Fis 1 expression in HD rats compared to SD rats (Fig. 8A-E). Mitophagy-mediated mitochondrial turnover is a key regulatory factor that maintains healthy mitochondria and has an induced effect on mtDNA copy number. Mitophagy genes Pink, Parkin, and Optn were measured in SD and HD kidney and found that HD have reduced gene expression for Optn, whereas Pink and Parkin demonstrated a significant increase in gene expression compared to SD control rats.The mitochondrial dysfunction associated with IR was identical in both normal and HD rats with significant alterations in the expression of genes regulating mitochondrial quality, such as mitophagy, mitofission, and fusion. These severely altered mitochondrial fission and fusion gene expressions during IR were restored by the STS pre-treatment in rats fed an SD and HD diet, except mitophagy where HD rats does not show any significant difference with respect to HD-IR (Fig. 8F-H).

## Discussion

Renal IR injury is a leading cause of AKI, characterized by renal dysfunction, inflammation, oxidative stress, and mitochondrial dysfunction [34]– [35]. HD exacerbates AKI and accelerates progression to CKD by disrupting mitochondrial function and energy metabolism [36]. Addressing renal IR injury in both normal and fat-modified kidneys is crucial. STS, a metabolite of hydrogen sulfide (H_2_S), is known for its antioxidant, anti-inflammatory, and metal-chelating properties, showing protective effects against IR injury in various organs, including the kidney [37–40]. Given its effectiveness in managing conditions associated with mitochondrial dysfunction, we investigated sodium thiosulfate (STS)—a validated treatment for cyanide poisoning and calciphylaxis—for its potential to alleviate IR injury in both standard and high-fat diet kidneys. Our study demonstrated that STS mitigates IR injury by reducing tissue damage, enhancing renal function, and restoring mitochondrial bioenergetics, including mitochondrial copy number and dynamics. These benefits were more pronounced in normal kidneys than in those exposed to a high-fat diet, highlighting the detrimental impact of diet-induced alterations on STS efficacy. Furthermore, STS alleviated mitochondrial bioenergetic decline and oxidative stress and improved mitophagy in normal kidneys, with these benefits significantly reduced in high-fat diet-altered kidneys.

Our preliminary investigations have highlighted the cardioprotective potential of sodium thiosulfate against ischemia-reperfusion injury, with its mechanism linked to the PI3K/Akt and Jak/STAT signaling pathways. These pathways not only modulate antioxidant and anti-inflammatory defences but also regulate mitochondrial functions and quality. Furthermore, we demonstrated sodium thiosulfate’s capacity to enhance bioenergetic function by contributing electrons to the electron transport chain complex, thereby preserving cardiac mitochondria in the ischemia-reperfusion experimental setting [20, 41]. In the present study, the beneficial effect of STS in ameliorating AKI induced by bilateral renal artery ligation for 45 min is in agreement with the reno-protective effect of STS reported by other investigators [42–44]. Furthermore, we discovered that the same dose of STS was beneficial, although to a lower extent, in treating renal IR in renal tissues that had been altered by high HD consumption for 16 weeks.

Oxidative stress is crucial in the development of renal diseases, accelerating the transition from AKI to CKD over time [45]. Renal IR produces reactive oxygen species (ROS) by disrupting mitochondrial function and causing excessive inflammation [1, 19]. Mitochondrial ROS generation is closely linked to cellular inflammation [19]. While antioxidants have been tested for ischemia-reperfusion treatment, their effectiveness has been variable, often due to poor mitochondrial accessibility [46–48]. Our study highlights sodium thiosulfate (STS) as effective in reducing oxidative stress in both standard and high-fat diet-fed groups by mitigating damage and boosting antioxidant defences [49].

Prior research showed that a high-fat diet can disrupt renal mitochondria and hinder recovery from IR injury [13, 14]. Mitochondria are crucial for adapting to energy imbalances [8]. In high-fat diet-fed kidneys, mitochondrial response to IR differs due to early functional changes [14]. High-fat diet can significantly alter renal mitochondrial function, particularly by impairing mitochondrial dynamics and bioenergetics [50]. The kidneys of HD-fed animals exhibit early mitochondrial dysfunction, including decreased mitochondrial membrane potential, impaired ATP production, and altered mitochondrial morphology [14, 50].

The reduced efficacy of STS in high-fat diet-altered kidneys can be attributed to several mechanistic differences in mitochondrial characteristics. High-fat diets induce lipid accumulation and lipotoxicity, which disrupt mitochondrial membrane integrity and impair electron transport chain (ETC) activity. This leads to reduced ATP production, thus limiting STS’s ability to restore mitochondrial function effectively [50]. Moreover, high-fat diets trigger metabolic reprogramming, including impaired sulfur metabolism, which compromises the ability of STS to exert its therapeutic effects. The altered mitochondrial dynamics in HD-exposed kidneys, characterized by disrupted fission and fusion processes, leads to the accumulation of dysfunctional mitochondria, further hindering STS’s impact on mitochondrial recovery [14]. The compromised mitophagy seen under HD conditions prevents the clearance of defective mitochondria, while suppressed PGC-1α signaling reduces mitochondrial biogenesis, limiting the formation of new functional mitochondria [14, 50]. Chronic low-grade inflammation and oxidative stress overload in HD conditions exacerbate mitochondrial dysfunction and contribute to fibrosis, further impairing the kidney’s ability to recover from IR injury [14].

Notably, while STS has been shown to enhance mitochondrial function by donating electrons to ETC complexes and restoring bioenergetic capacity, its protective effects are more pronounced in normal kidneys, where the mitochondrial environment remains relatively intact [20, 41]. In contrast, the hostile cellular environment in high-fat diet-fed kidneys, characterized by excessive ROS production and impaired mitochondrial dynamics, diminishes the efficacy of STS treatment. Excess ROS exacerbates oxidative damage to mitochondrial DNA, proteins, and lipids, limiting the capacity of STS to mitigate mitochondrial dysfunction [45].

Thus, while STS demonstrates potential in reducing oxidative stress and improving mitochondrial function, its therapeutic effect is limited in high-fat diet-modified systems due to the extensive mitochondrial dysfunction, oxidative stress, and chronic inflammation induced by the diet. Studies suggest that the nutrient composition and energy status of mitochondria are directly influenced by dietary factors, and high-fat diets disrupt these processes by affecting mitochondrial fusion and fission, ultimately impairing mitochondrial quality control and function [50]. Adequate nutrient levels are vital for mitochondrial function, with micronutrients playing key roles in energy metabolism and ATP production. While a high-fat diet impairs mitochondrial dynamics, interventions such as caloric restriction or ketogenic diets can have different effects. Caloric restriction reduces mitochondrial biogenesis but enhances mitophagy, a process that helps clear dysfunctional mitochondria. In contrast, ketogenic diets, while promoting mitochondrial biogenesis, show mixed effects on mitochondrial dynamics and mitophagy [51]. A more detailed understanding of the mechanistic differences in mitochondrial characteristics between standard and high-fat diet groups is crucial for optimizing STS treatment strategies in diet-induced metabolic disturbances and improving outcomes in conditions like AKI.

Future research should investigate the long-term effects of STS administration in high-fat diet models to better understand its potential as a therapeutic strategy for conditions like AKI and CKD. One possible experimental approach could involve administering STS for an extended period in HD-fed animals and assessing kidney function, mitochondrial health, and oxidative stress markers over time. This would help determine whether STS can provide sustained protection against ongoing metabolic disturbances or if repeated administration is necessary to maintain therapeutic effects. Additionally, examining the impact of STS on different stages of kidney injury, from acute to chronic phases, could offer valuable insights into its role in mitigating the progression of kidney disease. Hypotheses for such studies could include exploring whether STS administration can restore mitochondrial function and reduce fibrosis during the chronic stages of HD-induced renal damage or whether it can modify metabolic pathways to prevent the onset of irreversible kidney damage. These investigations will be crucial in refining STS-based treatments for metabolic kidney diseases and improving clinical outcomes.

## Conclusion

In conclusion, Renal IR injury is a major cause of acute kidney injury, complicating trauma, surgeries, and transplantation. While many pharmaceuticals have been used to mitigate its effects, their clinical success varies. Sodium thiosulfate (STS), known for its anti-ischemic properties in the heart, has not been previously studied in the kidneys. Our findings show that STS effectively reduces renal IR injury, enhances post-surgical recovery, and improves mitochondrial function and quality. Despite high-fat diet-induced mitochondrial changes and worsened AKI, STS still alleviates IR injury, though with slightly reduced effectiveness compared to standard diet-fed rats.

Few vegetables contain sulfur compounds that may include thiosulfates or their precursors. Cruciferous vegetables such as broccoli, Brussels sprouts, cauliflower, cabbage, and kale have glucosinolates that can be metabolized into thiosulfates. Garlic and onions contain sulfur compounds like allicin and alliin, which are precursors to thiosulfates, with garlic being particularly rich in sulfur. Direct thiosulfates are more commonly found in supplements or processed foods than in fresh produce. Including such diets during the rehabilitation can improve the post-surgical recovery in renal patients who experienced IR injury.

## Supplementary Information

Below is the link to the electronic supplementary material.

Supplementary Material 1

## Data Availability

The datasets generated and analysed during the current study are available from the corresponding author on reasonable request.

## Abbreviations

IR: Ischemia reperfusion
HD: High fat diet
AKI: Acute kidney injury
ATP: Adenosine triphosphate
Al/Cr: urinary albumin/creatinine ratio
CrCl: Creatinine clearance
PI3k: Phosphoinositide 3-kinases
Akt: Protein kinase B
GSK3β: Glycogen synthase kinase 3 beta
BUN: Blood urea nitrogen
MDA: Malondialdehyde
GSH: Reduced glutathione
GSSG: Oxidised glutathione
GPx: Glutathione peroxidase
SOD: Superoxide dismutase
GR: Glutathione reductase
NQR: NADH-oxidoreductase
SQR: Succinate-coenzyme Q reductase
QCR: QH2:cytochrome c oxidoreductase
COX: Cytochrome c oxidase
Pgc 1α: Peroxisome proliferator-activated receptor-gamma coactivator
Tfam: Transcription regulation factor A
Polg: Mitochondrial DNA polymeraseγ
Dnm 1: Dynamin-1
Fis 1: Mitochondrial fission protein 1
Mff: Mitochondrial fission factor
Mfn1: Mitofusin 1
Mfn 2: Mitofusin 2
Pink: PTEN- induced kinase 1
Optn: Optineurin
