# Supplementary material for: Effect of sodium thiosulfate on preventing renal ischemia-reperfusion injury in high-fat diet-fed rats: the role of renal mitochondrial quality
Source: Biol Res. 2025 Aug 18;58:56. doi: 10.1186/s40659-025-00636-z (PMC12359898; doi:10.1186/s40659-025-00636-z)
Supplement: Supplementary file 1 — Supplementary Material 1 [file 40659_2025_636_MOESM1_ESM.docx]

**Supplementary Table 1** Primer details of mitochondrial quality control genes

| **Gene** | **Sequence** |
| --- | --- |
| β-actin-F | GTGTGGTCAGCCCTGTAGTT |
| β-actin-R | CCTAGAAGCATTTGCGGTGC |
| Nd 1-F | CCACCGCGGTCATACGATTA |
| Nd 1-R | AGGGCTAAGCATAGTGGGGT |
| Pgc 1α -F | GAGGGACGAATACCGCAGAG |
| Pgc 1α -R | CTCTCAGTTCTGTCCGCGTT |
| Tfam-F | GTTGCTGTCGCTTGTGAGTG |
| Tfam-R | GTCTTTGAGTCCCCCATCCC |
| Polg -F | CTTTGGGCTCCAGCTTGACT |
| Polg -R | TGGAGAAAATGCTTGGCACG |
| Dnm1-F | TTGCCCTCTTCAACACTGAGC |
| Dnm1-R | ATGAAGCTGTCAGAGCCGTT |
| Fis 1-F | CCAGAGATGAAGCTGCAAGGA |
| Fis 1-R | TTCCTTGAGCCGGTAGTTGC |
| Mff-F | GAAAACACCTCCACGTGTGC |
| Mff-R | CTGCTCGGATCTCTTCGCTT |
| Mfn 1-F | TGACTTGGACTACTCGTGCG |
| Mfn 1-R | GGCACAGTCGAGCAAAAGTG |
| Mfn 2-F | CTCTGTGCTGGTTGACGAGT |
| Mfn 2-R | TCGAGGGACCAGCATGTCTA |
| Pink 1-F | TGTATGAAGCCACCATGCCC |
| Pink 1-R | TCTGCTCCCTTTGAGACGAC |
| Parkin-F | AGTTTGTCCACGACGCTCAA |
| Parkin-R | CAGAAAACGAACCCACAGCC |
| Optn-F | GGGTTTCCCAGAACCGACTT |
| Optn-R | AAGGTCCGCTTTCTCAAGCC |
